# AtPCa-Net: anatomical-aware prostate cancer detection network on multi-parametric MRI

**DOI:** 10.1038/s41598-024-56405-7

**Published:** 2024-03-08

**Authors:** Haoxin Zheng, Alex Ling Yu Hung, Qi Miao, Weinan Song, Fabien Scalzo, Steven S. Raman, Kai Zhao, Kyunghyun Sung

**Affiliations:** 1 Radiological Sciences, University of California, Los Angeles, Los Angeles, 90095 USA; 2 Computer Science, University of California, Los Angeles, Los Angeles, 90095 USA; 3 Electrical and Computer Engineering, University of California, Los Angeles, Los Angeles, 90095 USA; 4 The Seaver College, Pepperdine University, Los Angeles, 90363 USA

**Keywords:** Cancer imaging, Urological cancer, Prostate, Cancer imaging, Urological cancer, Prostate

## Abstract

Multi-parametric MRI (mpMRI) is widely used for prostate cancer (PCa) diagnosis. Deep learning models show good performance in detecting PCa on mpMRI, but domain-specific PCa-related anatomical information is sometimes overlooked and not fully explored even by state-of-the-art deep learning models, causing potential suboptimal performances in PCa detection. Symmetric-related anatomical information is commonly used when distinguishing PCa lesions from other visually similar but benign prostate tissue. In addition, different combinations of mpMRI findings are used for evaluating the aggressiveness of PCa for abnormal findings allocated in different prostate zones. In this study, we investigate these domain-specific anatomical properties in PCa diagnosis and how we can adopt them into the deep learning framework to improve the model’s detection performance. We propose an anatomical-aware PCa detection Network (AtPCa-Net) for PCa detection on mpMRI. Experiments show that the AtPCa-Net can better utilize the anatomical-related information, and the proposed anatomical-aware designs help improve the overall model performance on both PCa detection and patient-level classification.

## Introduction

Prostate cancer (PCa) is the second leading cancer-related cause of death and the most common cancer among men in the United States^1^. Multi-parametric MRI (mpMRI) is the preferred non-invasive imaging tool for PCa diagnosis before biopsies^2,3^. According to the Prostate Imaging Reporting and Data System, version 2.1 (PI-RADS)^2,3^, a combination of mpMRI findings is used for predicting the probability of clinically significant PCa, where the different combinations are used depending on the lesion location, in either transition zone (TZ) or peripheral zone (PZ) of the prostate. For example, following the PI-RADS, T2-weighted imaging (T2WI) is the primary imaging component for lesions in TZ with an additional assessment by diffusion-weighted imaging (DWI), while in the PZ, DWI/apparent diffusion coefficient (ADC) is the essential imaging component with an addition of dynamic contrast-enhanced (DCE) MRI^2,3^. Therefore, given the significance of varying imaging appearances of PCa on mpMRI between TZ and PZ, there is considerable potential to improve PCa detection models further when this anatomical prior is thoughtfully incorporated.

With advances in deep learning, many studies proposed deep learning models for the detection of PCa using mpMRI. However, the different appearances of PCa lesions in TZ and PZ on different mpMRI components were generally not fully integrated into the model design^4–10^. Overlooking this zonal-related anatomical prior, but treating all lesions equally regardless of the locations, could lead to potential suboptimal model performance^2,3^. A design that can reflect both the zonal appearance differences and the commonality of them being PCa lesions is the key to improving the model’s performance.

Hierarchical label and loss design embed structural information hierarchically among different classes into the loss function to better guide the model training^11–13^. The design transforms the binary labeling to a more structured label space and is able to account for the distinct inter-class property differences while preserving shared properties among different classes^11–13^. In this study, we propose an anatomical-aware hierarchical loss design, the Zonal Loss (ZL). The ZL can direct the model to learn both the unique and shared characteristics of PCa lesions across different prostate zones in accordance with clinical practice, thus enhancing the model’s detection capabilities.

**Figure 1:**
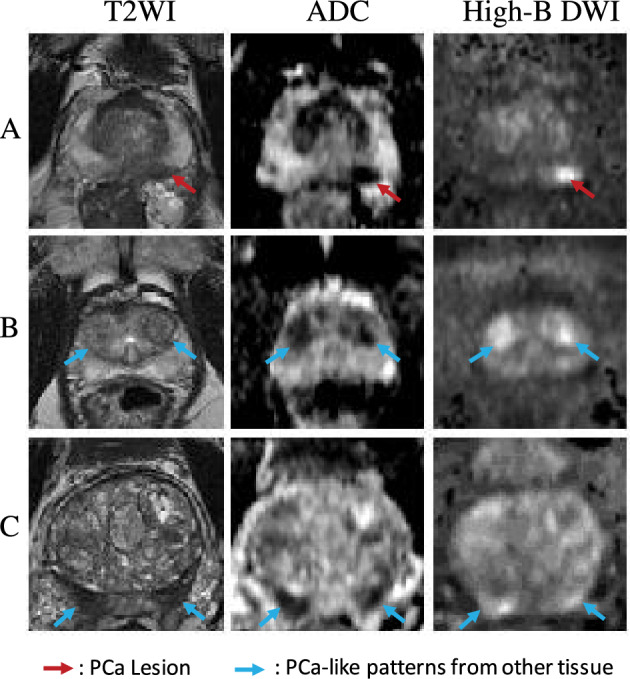
Examples of a PCa lesion and PCa-like patterns from other prostate tissues, like the BPH and the CZ, that can cause FP predictions from three mpMRI scans (A, B, and C). From the left to the right, the first column shows the T2WI images, the second column shows the ADC images, and the third column shows the high-B DWI images. Red arrows point to a PCa lesion, and blue arrows point to the PCa-like patterns in BPH (B) and CZ (C).

Furthermore, studies have shown that PCa, benign prostatic hyperplasia (BPH), and the central zone (CZ) of the prostate can occasionally present with visual similarities^14,15^. This undesirable resemblance between PCa and other prostate tissues complicates the diagnosis process. In clinical practice, symmetric-related information as a reference is valuable for distinguishing BPH and CZ from PCa. Research indicates that BPH and CZ tend to be visually symmetric^14,15^, while PCa is generally presented asymmetrically^16,17^. Illustrations of PCa lesions and PCa-like visual patterns are shown in Fig. 1. We can observe that BPH and CZ are shown to be similar to PCa lesions-low intensity on both ADC and T2WI images and high intensity on high-B DWI images.

The visual similarity of non-PCa prostate tissues not only complicates the diagnostic process but also leads to performance degradation in PCa detection models due to the generation of undesired false positive (FP) predictions-a common issue in existing deep-learning-based PCa detection models^4–10^. By taking symmetric-related patterns into consideration, FP predictions may be reduced as PCa lesions can be further distinguished from BPH and CZ by their asymmetrical appearance differences. Therefore, integrating symmetry-related anatomical priors into the design of PCa detection models may be crucial in reducing potential FP predictions.

Existing study has shown that the human visual system recognizes symmetric patterns by comparing visual differences between the original image and the mirrored image after reflecting with respect to an imagined center-axis^18^. Inspired by how symmetric patterns stimulate human visual perception, we propose a symmetric-aware network architecture that utilizes both original and mirrored mpMRI images for PCa detection. Simulating how the human vision system reacts to the symmetric patterns, the network can help distinguish the PCa lesions from other prostate tissue with visual similarities, like BPH and CZ, and thus reduce FP predictions^14–17^.

In this study, we introduced PCa-related anatomical priors into the deep learning framework design and developed an anatomical-aware deep learning network for PCa detection on mpMRI. The proposed network leverages symmetry-related information and PCa zonal appearance differences on mpMRI images to form a 3D anatomically-aware PCa detection network (AtPCa-Net), enhancing the accuracy of PCa lesion detection. Our main contributions include the following:

1. We exhibit that the introduction of the PCa-related anatomical priors into the DL network architecture design helps improve model performance. The extensive experiments demonstrate that either one of the anatomical-aware designs of the proposed AtPCa-Net can help improve the PCa detection and patient-level classification performance, and the integration of both designs can achieve the best model performance on both PCa detection and patient-level classification tasks.
2. We incorporate the symmetric-related clinical priors into the network architecture design to suppress potential FP predictions. By utilizing symmetric-related visual appearance differences, the design could help distinguish PCa from other prostate tissues shown similar visual patterns on mpMRI, thereby reducing possible FP predictions. To the best of our knowledge, our study is the first study to achieve FP reductions on PCa detection using anatomical-related clinical prior.
3. We integrate the zonal appearance differences of PCa on mpMRI explicitly into the loss design by proposing the Zonal Loss (ZL). Compared with existing models overlooking this property and treating all lesions equally regardless of their location, the ZL treats PCa in different prostate zones differently following the clinical guideline, and thus helps improve model performance.
4. Compared with other baselines, the AtPCa-Net achieved lower FP predictions while maintaining same sensitivity. Although the model still needs to be improved in order to be deployed in clinical practice, the results suggests the potential to further reduce the number of unnecessary target biopsies could be caused by using current computer-aided diagnosis models.

## Related works

### Prostate cancer detection

The deep-learning models for PCa detection and classification based on mpMRI have been widely investigated^19–22^. The models are generally built by convolutional neural networks (CNNs) for their outstanding performance on classification, segmentation, and detection tasks. Recent studies exhibit the feasibility of using CNNs for PCa detection using mpMRI^4–10,23^. Li et al.^23^ designed a multi-scale two-branch dilated-convolution-based deep learning network to segment both PCa lesions and prostate from mpMRI. Seetharaman et al.^4^ designed a PCa detection network to identify indolent and aggressive PCa separately with the help of two different encoder branches for T2WI and ADC images and the fusion of feature maps from multiple levels of the encoder branches. Cao et al.^8^ introduced the idea of ordinal encoding for PCa with different severity doing a multi-class classification. The author also designed the mutual finding loss mimicking the process of how radiologists interpret the T2WI and ADC images to detect PCa. Cao et al.^9^ modified the FocalNet^8^ to have a stack of adjacent slides as input and did a comprehensive evaluation of the PCa detection performance between the radiologists and their proposed model.

There are some existing studies that tried to integrate clinical priors related to different diagnostic focuses for PCa in different zones into the PCa detection network design^3,6^. Hosseinzadeh et al.^5^ utilized this zonal-related anatomical prior by stacking the prostate zonal segmentation masks together with mpMRI images as part of the input to the model and showed the detection performance improved. Vente et al.^6^ discovered the idea of both using ordinal encoding for PCa with different aggressiveness and also feeding prostate zonal masks into the PCa detection networks to let the model learn the anatomical relationship between the prostate zones and PCa appearance. Duran et al.^24^ discussed the performance differences between using a prostate mask or using a PZ mask as part of the segmentation model input and observed the latter approach got better lesion segmentation performance.

Although they provide zonal information from the input, the cross-entropy (CE) loss with binary labeling they used explicitly treated all lesions identically regardless of the location^2,3^. The ignorance of the lesion appearance differences in different prostate zones might lead to suboptimal PCa detection performance, which could be further improved.

As PCa detection models generally suffer from undesired FP predictions, some studies have introduced network designs aiming for better FP reduction ability^7,10^. Yu et al.^7^ proposed a multi-scale patch-wise network together with a squeeze-and-excitation (SE) block^25^. The design tried to reduce FP predictions by letting the model learn the FP patterns from the context information provided by the multi-scale patches automatically. To suppress FP predictions, Saha et al.^10^ first introduced an auxiliary network to classify if a given image patch contains PCa lesions or not and then multiplied the classification results with the detection probability map to conduct the final output. The experiments showed that the FP predictions could be suppressed by the patch-wise classification results. However, neither study has considered achieving FP reduction using anatomical-related clinical prior, which could also be capable of helping effectively reduce FP predictions.

### Anatomical-aware design for other diseases

There are existing studies investigating how to incorporate anatomical-related clinical priors into the network architecture design for various tasks related to other diseases^26–28^. Sun et al.^26^ introduced a weakly-supervised knowledge distillation model for breast mass segmentation, with auxiliary networks for reconstruction and aggressiveness classification. The anatomy property was designed to be learned from the encoder of the teacher model, an autoencoder network reconstructing the input image, and then transferred to the student model, the desired breast mass segmentation network. Kamal et al.^27^ proposed a semi-supervised CNN for thoracic disease classification on chest Chest X-ray images. The anatomical information was brought by the prediction masks of lung and heart generated from the auxiliary segmentation network and then fed into the main classification network as an anatomy-informed reference for an attention module. Ma et al.^28^ proposed a dual-branch cascaded CNN for the segmentation of retinal layers and fluid from optical coherence tomography (OCT) images. The model first calculated a relative positional map based on the retinal layer boundaries and then fed them into the final segmentation network to inform the model of the anatomical relationships among different retinal layers. All the studies showed improvements in model performance when including anatomical-aware network architecture design. In this study, the anatomical-aware designs are not only composed of a symmetric-aware network architecture for FP prediction reduction but also shown through the design of the hierarchical loss, the ZL, considering the diagnostic differences of lesions on different prostate zones following clinical guideline^2,3^.

## Methods

### Overview

**Figure 2:**
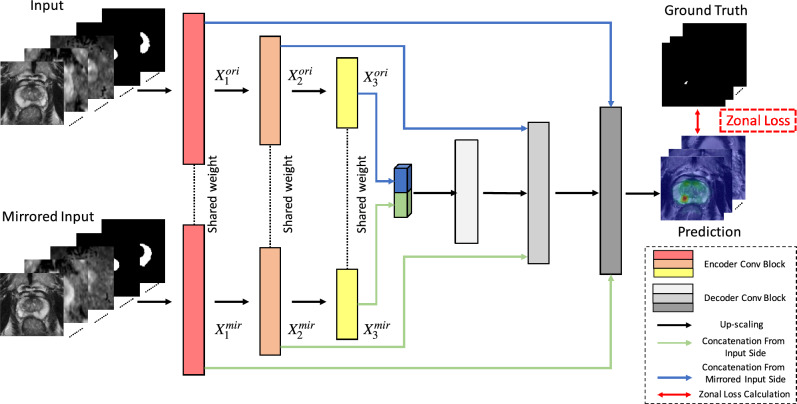
Architecture of the proposed AtPCa-Net. It combines the 3D symmetric-aware network and the proposed Zonal Loss (ZL). The network takes input stack images of T2WI, ADC, high-B DWI, TZ’s mask, and PZ’s mask images in an original way and a mirrored way. The weights of encoders at each level from the two sides of the figure are shared. At each level, the feature maps from two sides are concatenated first together and go through a bridge convolution block that consists of two consecutive 3D convolution blocks, then concatenated with the upscaled feature maps from the lower level, and finally upscaled to the upper-level decoder layers.

We propose a 3D anatomical-aware PCa detection network (AtPCa-Net) to detect whole-mount histopathology (WMHP) confirmed clinically significant PCa (csPCa) utilizing the PCa-related anatomical priors. The proposed AtPCa-Net consists of two parts. First, a 3D symmetric-aware network takes the symmetric-related information into consideration to suppress FP predictions. Second, the ZL structurally integrates the PCa-related zonal differences into the label and loss design. The overall architecture of AtPCa-Net is illustrated in Fig. 2. We adhered to the structure of nnU-Net as the backbone for AtPCa-Net because of its good performance on detection and segmentation of medical imaging tasks^29^.

### Dataset

#### Study population and mpMRI images

This retrospective study was carried out in compliance with the United States Health Insurance Portability and Accountability Act (HIPAA) of 1996 with approval from the institutional review board (IRB) of our institution with a waiver of the requirement for informed consent. All experiments conducted in this study adhered strictly to the relevant guidelines and regulations. The whole dataset consists of 652 patients. It is composed of two parts: (1) pre-operative mpMRI images from patients (N = 220) who had confirmed PCa lesions (N = 246) with whole-mount histopathology after radical prostatectomy (RP), and (2) mpMRI images from patients (N = 432) who did not have indications of PCa lesions, confirmed by systematic biopsies followed by negative mpMRI (PI-RADS$\le$2). We included mpMRI images with no indications of PCa lesions to balance the data distribution on model training and testing, as well as to perform patient-level classification evaluation. We used 5-fold cross-validation to validate and evaluate the model performance, in which each fold contains 130/131 patients assigned randomly from the entire dataset.

All mpMRI images are performed on Siemens 3T scanners with the standardized clinical prostate mpMRI protocol^2,3^, including T2WI and DWI. We exclude the DCE-MRI images given the limited role of DCE-MRI^2,3,30,31^. For T2WI, the repetition time (TR) and echo time (TE) are 3000–5900 ms and 101–109 ms, the field of view (FOV) of 20 cm $\times$ 20 cm with an in-plane resolution of 0.625 mm $\times$ 0.625 mm and through-plane resolution of 3 mm. For DWI, we use TR and TE of 4800–5300 ms and 60–81 ms, FOV of 26 cm $\times$ 21 cm with in-plane resolution of 1.625 mm $\times$ 1.625 mm and through-plane resolution of 3.6 mm. The ADC maps were calculated using linear least squares curve fitting of voxels in the four DWIs against their corresponding b values (0/100/400/800 s/mm2 ). We also denote the DWI images with b = 1400 s/mm2 as high-B value DWI (high-B DWI).

#### Clinical interpretation and annotations

The mpMRI images were reviewed by three genitourinary (GU) radiologists (10+ years of clinical prostate MRI reading) as part of the standard clinical procedure following the clinical guideline^2,3^. Lesion findings with PI-RADS score $\ge$ 3 are reported as MRI-positive findings, and the findings with PI-RADS score < 3 are interpreted as MRI-negative findings in this study.

The ground truth of the lesion annotations is confirmed by WMHP after RP matched to mpMRI prior to RP in this study. Blinded to all MRI-related information, the sliced WMHP specimens are examined and reported by three GU pathologists (with 14, 8, and 5 years of experience in clinical prostate histopathology interpretation) as part of the standard clinical procedure. Every PCa lesion was contoured and assigned a corresponding Gleason Score (GS) on WMHP. PCa lesions with GS$\ge 7$ are defined as csPCa and are the detection targets of our proposed detection model in this study.

GU radiology research fellows, under the supervision of GU radiologists, retrospectively reviewed each mpMRI exam and contoured the region of interest (ROI) of MRI-visible lesions on T2WI images referring to the WMHP examination reports. MRI-positive findings are categorized as true positive if the radiological findings and the pathological findings are matched or false positive if no corresponding PCa lesion is found in histopathology reports. We defined the prospectively missed lesions that are retrospectively identified in the re-review procedure as false-negative (FN) lesions. The remaining PCa lesions that are MRI non-visible and also retrospectively unidentifiable on mpMRI are not included in the study as we cannot accurately contour them.

Compared to data consisting of biopsy-confirmed PCa, ground truth confirmed by WMHP offers additional insights into how the model would react to FN cases, which are generally harder to recognize in clinical practice. Understanding FN lesions is crucial, as overlooked or underestimated PCa can lead to insufficient treatment and undesired oncological outcomes^32,33^.

The prostate zonal segmentation of TZ and PZ are treated as part of the AtPCa-Net’s input, shown in Fig. 2. The zonal masks are generated using a separate automatic prostate zonal segmentation model, CAT-Net^34^, to explicitly provide the PCa-related anatomical information.

#### Preprocessing

The T2WI images underwent N4 bias filed correction to compensate for the low-frequency intensity non-uniformity^35^. The high-B DWI and ADC images are registered and resampled with respect to T2WI images using rigid spatial transformation while utilizing real-world coordinates information for each patient since the DWI and T2WI sequences are acquired temporally closed and only minimal patient motion are found^8,36,37^. After the registration, high-B DWI, ADC, and T2WI images are rotated with respect to the center line, generated by connecting the volumetric center of the prostate and the TZ, to show the symmetric appearance. Then, high-B DWI, ADC, and T2WI images are center cropped with the size of 128 $\times$ 128 pixels from the original 320 $\times$ 320 pixels images as all prostates are allocated in the center of the acquired MR images following the clinical protocol^2,3^. The intensity value of voxels in high-B DWI and T2WI images are linearly normalized to have a value in the range of [0, 1]. As the values of ADC maps are quantitative, the voxel intensities are consistent across patients^2,3,8,38^. Therefore, the intensity on ADC maps is first clipped by a patient-independent value and then normalized to be in the range of [0, 1]^8^.

### Symmetric-aware network architecture

We first introduce the proposed symmetric-aware network architecture design that is capable of taking symmetric information into consideration explicitly. The detailed network architecture can be seen in Fig. 2. In this study, we implement a UNet-like backbone structure since the UNet-like structures have shown great performances in medical-imaging-related segmentation and detection tasks^29^. The inputs are the 3D volumetric stack of images with a dimension of [$N \times C_{in} \times D_{in} \times H_{in} \times W_{in}$], where *N* is the batch size, $C_{in}$ is the number of channels, $D_{in}$ is through-plane resolution, $[H_{in}, W_{in}]$ are in-plane resolution of the input mpMRI images. Different categories of images (T2WI, ADC, high-B DWI, binary mask of TZ, or binary mask of PZ) correspond to different channels of the input, and each imaging modality has the same volumetric size of $[D_0, H_0, W_0]$. The network takes two inputs: one is the original 3D stack of images, and the other is the 3D stack of images mirrored across the vertical axis.

The weights of all the convolution blocks (ConvBlock) in each encoder layer of the network are shared by both the original and mirrored paths. The design of shared-weight encoders has proven to be useful when visual comparisons are applied in downstream tasks in the DL network architecture designs^39^, similar as how the symmetric-related anatomical priors were used to distinguish between benign and cancerous prostate tissue in the clinical practice. By sharing weights on the two encoders, features extracted from both pathways will maintain symmetric information. This, in turn, assists the network in learning how to utilize these symmetric features, resembling the human visual system, and thus enhances the model’s decision-making process. In each level *i* other than the bottom level, the extracted feature maps $X_{i}^{ori}\in$ [$N \times C_{i} \times D_{i} \times H_{i} \times W_{i}$] and $X_{i}^{mir}\in$ [$N \times C_{i} \times D_{i} \times H_{i} \times W_{i}$] from both sides will be first concatenated channel-wisely and format a combined feature map $X_{i}^{cat}\in$ [$N \times 2C_{i} \times D_{i} \times H_{i} \times W_{i}$]. Then $X_{i}^{cat}$ will pass through a bridge block (BridgeBlock), composed of two 3D convolution blocks, and finally concatenated together with the upscaled feature map $X_{i+1}^{up}$ from level $i+1$. The final concatenation will then be used to do further feature extraction at that level. Detail representations can be seen in the sub-figure of Decoder Blocks in Fig. 2.

The output of the network is the detection probability map of where suspicious csPCa is allocated. The difference between the probability map and the ground truth mask is measured by the proposed ZL, which will be introduced in the following sub-section.

### Zonal loss

Current labeling strategies in PCa detection models generally inadequately account for the significance of PCa’s zonal appearance differences, but using CE loss treating all PCa lesions identically regardless of their location^2–10^. We propose an anatomical-aware hierarchical label and loss design, the ZL, to guide the model to learn the different appearances of PCa lesions in different zones with anatomy-informed constraints.

We denote the set of voxels of the PZ region as ${\mathbb {P}}$, the TZ region as ${\mathbb {T}}$, and the csPCa lesion region as ${\mathbb {L}}$ for a given prostate mpMRI image. Given an input image ${{\mathscr {I}}} \in {\varvec{R}}^{\ C\times D\times H\times W}$ and the corresponding binary mask ${{\mathscr {M}}} \in \{0, 1\}^{D\times H\times W}$, where $C,\ D,\ H,\ W$ are the channel number, depth, height and width of the input image ${{\mathscr {I}}}$, for any voxel $v \in {{\mathscr {I}}}$, the corresponding label voxel *m* on ${{\mathscr {M}}}$ in binary CE loss design is given as:

1 $$\begin{aligned} m=\left\{ \begin{array}{ l l } 1 &{} \quad \text {if }v\in {\mathbb {L}},\\ 0 &{} \quad \text {otherwise} \end{array} \right. \end{aligned}$$

One of the key points of the hierarchical label and loss design is the multi-level labeling design with respect to the number of properties each class holds—lower-level classes hold fewer properties and constraints, higher-level classes hold more properties and more constraints correspondingly^11–13^. According to PI-RADS^2,3^, PCa lesions in PZ mostly require visual information related to DWI, while lesions in TZ require a combined evaluation of both T2WI and DWI for accurate diagnosis. We adopt this clinical interpretation process using the hierarchical label and loss design by treating the class of TZ lesions as requiring additional information from T2WI images compared with the class of lesions in PZ for improved PCa detection on mpMRI. Hence, the ZL design is adept at acknowledging the distinct zonal appearance of PCa while preserving the anatomical congruence between lesions in the TZ and PZ.

We design a hierarchical labeling with ground truth mask ${{\mathscr {M}}} \in \{0, 1\}^{2\times D\times H\times W}$ for a given image ${{\mathscr {I}}} \in {\varvec{R}}^{\ C\times D\times H\times W}$. For any voxel $v \in {{\mathscr {I}}}$ , the corresponding label vector ${\varvec{m}}=[m_0, m_1]\in \{0,1\}^2$ in ${{\mathscr {M}}}$ in our loss design is given as:

2 $$\begin{aligned} {\varvec{m}} = {\left\{ \begin{array}{ll} {[}1, 1], \ \ \ \ \text {if}\ v\in {\mathbb {L}}\cap {\mathbb {T}}\\ {[}1, 0], \ \ \ \ \text {if}\ v\in {\mathbb {L}}\cap {\mathbb {P}}\\ {[}0, 0], \ \ \ \ \text {otherwise} \end{array}\right. } \end{aligned}$$

This label design aims to adopt the clinical prior knowledge to the detection of csPCa lesions. Abnormalities should be observed on image sequences related to DWI in common for both the lesions in TZ and PZ ($m_0$), and additional abnormal observations from T2WI are needed for lesions in TZ in order to make more accurate diagnoses ($m_1$)^2,3^.

We denote the probability vector ${\varvec{p}}=[p_0, p_1]\in [0,1]^2$ in the output probability map ${{\mathscr {P}}}\in [0,1]^{2\times D\times H\times W}$, for the corresponding voxel $v \in {{\mathscr {I}}}$. The modified CE loss can be written as:

3 $$\begin{aligned} \begin{aligned} {\mathscr {L}}({{\mathscr {P}}}, {{\mathscr {M}}})&= \sum \limits _{v}-{\varvec{m}} \log {{\varvec{p}}}-({\varvec{1}}-{\varvec{m}})\log {({\varvec{1}}-{\varvec{p}})} \end{aligned} \end{aligned}$$

where $${\textbf{1}} = [1,1]$$, and:

4 $$\begin{aligned} p_0= & {} \left\{ \begin{array}{ l l } p_0\ {} &{}\text {if}\ m_0\ = 1\\ max(p_0, p_1) &{}\text {if}\ m_0\ = 0 \end{array} \right. \end{aligned}$$

5 $$\begin{aligned} p_1= & {} \left\{ \begin{array}{ l l } min(p_0, p_1)\ {} &{}\text {if}\ m_1\ = 1\\ p_1 &{}\text {if}\ m_1\ = 0 \end{array} \right. \end{aligned}$$

The modified CE loss is designed to suppress prediction vector patterns that should not exist. Based on our labeling design, label vector pattern $\hat{{\varvec{m}}}=[0, 1]$ is not defined, since solely abnormalities found on T2WI have limited contribution to the diagnosis of suspicious PCa^2,3^. Therefore, any output probability vectors with $p_1>p_0$ should be penalized in order to teach the model not to conduct such predictions. However, the original CE loss with binary labeling only computes the loss of each class independently but ignores this inter-class relationship. We intentionally add this constraint onto the original CE loss, which is shown in (4) and (5). In (4), $p_0=max(p_0, p_1)$ when $m_0=0$, and in (5), $p_1=min(p_0, p_1)$ when $m_1=1$ both indicate that $p_0$ should be greater than $p_1$ in any prediction outputs, and any patterns disobey this rule should be penalized. This modification could further help the model converge to a better solution.

In addition, we adopt Focal Loss onto the modified CE loss in Eq. (3) to account for the imbalance in the number of voxels between the csPCa lesions and background^40^. This would reduce the relative weight for well-classified voxels and emphasize focus on hard ones like lesion voxels^8,40^. The final ZL form follows:

6 $$\begin{aligned} \begin{aligned} {\mathscr {L}}^{ZL}({{\mathscr {P}}},{{\mathscr {M}}}) = \sum \limits _{v}-&{\varvec{m}}({\varvec{1}}-{\varvec{p}})^{\gamma }\log {{\varvec{p}}}- ({\varvec{1}}-{\varvec{m}}){\varvec{p}}^{\gamma }\log {({\varvec{1}}-{\varvec{p}})} \end{aligned} \end{aligned}$$

where ${\textbf{1}} = [1,1]$, and ${\varvec{p}}\in [0,1]^2$ is defined in (4) and (5).

### Implementation details

In each of the three levels of the network architecture, the channel number is [64, 128, 256] for each level of the convolutional layers of the encoders, and [256, 128, 64] for each level of the convolutional layers in the decoders, correspondingly^29^. Each level of the convolutional layers comprises four consecutive ConvBlocks, and each ConvBlocks consists of a $3\times 3\times 3$ 3D convolution kernel, following by a LeakyReLU activation function and an instance normalization, following the settings of the nnU-Net^29^.

Each training procedure takes 60 epochs, with early-stopping strategy applied when no loss degradation for 30 accumulate epochs was found to avoid potential overfitting issues. Adam optimizer^41^ was adopted with the loss function of the Focal Loss^40^ by default, the ZL when specifically stated. All models are trained on an Nvidia RTX3090 GPU.

## Results

### Quantitative results

For csPCa lesion detection, we evaluate the overall csPCa detection performance of the AtPCa-Net using the free-response receiver operating characteristic (FROC) analysis^20^. The FROC curve helps analyze the relationship between model detection sensitivity and the level of FP predictions per patient. In the experiment, we consider the local maxima on the output probability map as the csPCa detection points. The csPCa detection point is defined as a true positive (TP) when the point is within 5 mm of any csPCa ground truth ROIs to account for a potential mismatch between the whole-mount specimen and mpMRI of the corresponding ROI^8,20^.

We also evaluate the per-patient level classification performance of the proposed AtPCa-Net by defining patients with csPCa as positive cases, and patients without csPCa as negative cases. For each patient, we treat the highest value on the output probability map as its probability of having csPCa. The evaluation of the per-patient level classification performance is done by using Receiver Operating Characteristic (ROC) analysis. In both ROC and FROC analysis, we evaluate the model performances by 5-fold cross-validation after 1000 times bootstrapping. ROCs were compared with DeLong Test^42^, and the sensitivity results at each number of FP predictions per patient were compared using Chi-squared Test, in accordance with 95% confidence interval (95% CI), correspondingly.

**Figure 3:**
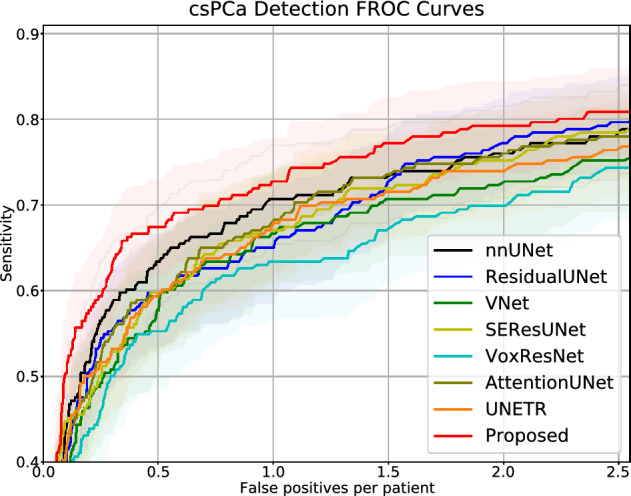
csPCa detection performance comparison via FROC curves, which measured by the detection sensitivity (y-axis) against number of false-positive predictions per patient (x-axis). Solid lines are the mean FROC curves, and shadow areas represent the corresponding 95% confidence interval.

**Figure 4:**
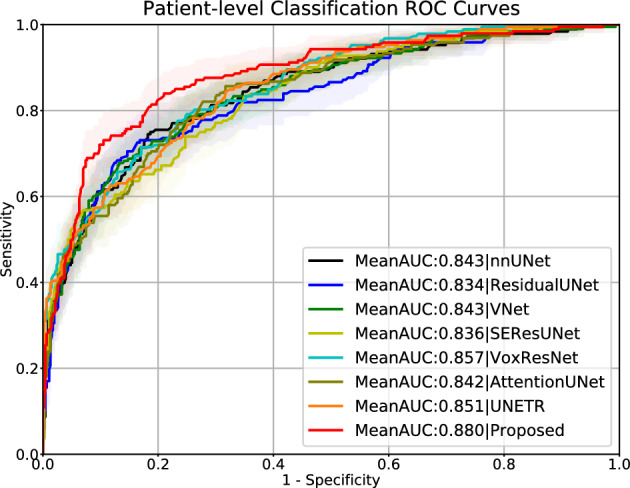
Patient-level classification performance comparisons via ROC curves, which are measured by the sensitivity (y-axis) against the false-positive rate (x-axis). Solid lines are the mean ROC curves, and shadow areas represent the corresponding 95% confidence interval.

We performed comparisons between our proposed model and other popular 3D image segmentation models, including SEResUNet^25^, ResidualUNet^43^, VNet^44^, AttentionUNet^45^, VoxResNet^46^, nnUNet^29^, and UNETR^47^ for csPCa detection and patient-level classification. Figure 3 visualizes the comparison of csPCa detection performance among different models via different FROC curves. Figure 4 visualizes the comparison of patient-level classification performance among different models via ROC curves. Table 1 shows the comparisons of the patient level classification AUCs in the format of mean, and the comparisons of csPCa detection performance via showing the sensitivity results against 0.5/1/1.5/2/2.5 FP predictions per patient. Our proposed AtPCa-Net outperforms all other models on all the FROC measurements on 0.5/1/1.5/2/2.5 FP predictions per patient with higher mean sensitivities (p<0.05). The AtPCa-Net also outperforms all other models on the patient-level classification AUCs (p<0.05).

Compared with some of the existing studies proposing PCa detection using PCa biopsy results^4,5,37^, our study uses results confirmed by WMHP, which results in additional FN csPCa annotations as the prospectively missed csPCa lesions were retrospectively annotated. In order to discuss the possible performance discrepancies caused by the dataset’s differences between existing studies and ours, we also perform ROC and FROC analysis to the results using the dataset after excluding FN lesions, shown in Table 5. The proposed AtPCa-Net outperforms all other baseline models^25,43–47^ on both ROC and FROC measurements when using the dataset excluding all FN lesions (p<0.05), which keeps consistent to its performance when including FN lesions, as shown in Table 1.

**Table 1:** Patient-level classification and csPCa detection performance comparisons among different models.

Models	Patient classification AUC (%95 CI)	csPCa detection sensitivity (95% CI)				
		0.5 FP/Patient	1 FP/Patient	1.5 FP/Patient	2 FP/Patient	2.5 FP/Patient
nnUNet	0.843 (0.803, 0.883)	0.634 (0.571, 0.697)	0.707 (0.651, 0.763)	0.736 (0.684, 0.788)	0.760 (0.707, 0.813)	0.789 (0.735, 0.853)
ResidualUNet	0.834 (0.794, 0.874)	0.595 (0.534, 0.656)	0.650 (0.592, 0.708)	0.727 (0.674, 0.780)	0.772 (0.724, 0.820)	0.797 (0.745, 0.849)
VNet	0.843 (0.801, 0.885)	0.594 (0.529, 0.659)	0.667 (0.604, 0.730)	0.707 (0.644, 0.770)	0.728 (0.667, 0.789)	0.752 (0.696, 0.808)
SEResUNet	0.836 (0.797, 0.875)	0.598 (0.534, 0.662)	0.671 (0.609, 0.733)	0.720 (0.656, 0.784)	0.752 (0.692, 0.812)	0.785 (0.728, 0.842)
VoxResNet	0.857 (0.824, 0.890)	0.553 (0.489, 0.617)	0.634 (0.572, 0.696)	0.675 (0.615, 0.735)	0.699 (0.643, 0.755)	0.744 (0.683, 0.805)
AttentionUNet	0.842 (0.804, 0.880)	0.594 (0.532, 0.656)	0.683 (0.624, 0.742)	0.736 (0.677, 0.795)	0.760 (0.708, 0.812)	0.781 (0.732, 0.830)
UNETR	0.851 (0.815 , 0.887)	0.598 (0.537, 0.659)	0.679 (0.619, 0.739)	0.715 (0.659, 0.771)	0.740 (0.685, 0.795)	0.769 (0.716, 0.822)
AtPCa-Net (Proposed)	**0.880 (0.846, 0.914)**	**0.675 (0.620, 0.730)**	**0.728 (0.674, 0.782)**	**0.772 (0.716, 0.828)**	**0.793 (0.741, 0.845)**	**0.809 (0.757, 0.861)**

### Qualitative Results

**Figure 5:**
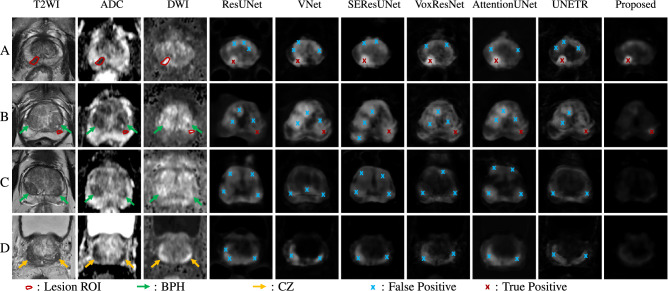
Visualizations of csPCa detection results among different models ResUNet^43^, VNet^44^, SEResUNet^25^, VoxResNet^46^, AttentionUNet^45^, UNETR^47^ on two patients with csPCa (A and B) and two without csPCa (C and D). From left to right, the first column shows the T2WI images, the second column shows the ADC images, the third column shows the high-B DWI images and all other columns show the detection probability maps generated by different models for each patient A, B, C and D correspondingly. Red contours shown on T2WI, ADC, and high-B DWI images indicate the contour of csPCa lesions. Yellow arrows point to the regions of CZ, and Green arrows point to the regions of BPH on the MR images. Blue crossings represent FP predictions, and red crossings represent TP predictions.

We qualitatively evaluate the model performance by showing representative examples of csPCa detection performance comparisons in Fig. 5. In Fig. 5, A and B correspond to two patients with csPCa, and C and D correspond to two patients without csPCa. Overall, the proposed AtPCa-Net conducted fewer FP predictions with the same TP predictions on all cases compared with other models^25,43–47^.

We can also observe its ability to suppress symmetric FP predictions with its better ability to distinguish symmetric abnormal patterns of csPCa from other normal prostate tissue, like BPH and CZ, compared with other models^25,43–47^. Patients B and C are representative examples of patients who have BPH, and Patient D is a representative example of patients whose CZ’s appearance could mislead the model’s prediction. The BPH, pointed by green arrows for Patient B and C on the MR images, and the CZ region, pointed by the yellow arrows for Patient D, show visually similar appearances as the csPCa on mpMRI images but with symmetric patterns. We can observe that for all patients, other models that do not take the symmetric-related anatomical information into consideration misidentify the BPH and the CZ as csPCa and result in FP predictions. Our models can correctly detect the csPCa with fewer FP predictions with the help of the symmetric-related anatomical-aware architecture design.

#### Backbone network extension

To show the generalizable potential of the proposed anatomical-aware design, we also try to transplant the architecture onto another UNet-like backbone network. We implement the nnUNet++^34^, a UNet++^48^ variant, with the proposed ZL and symmetric-aware architecture. Similar to the nnUNet-based approach, the weights of each non-decoder block are shared. In nnUNet++, the feature maps from both sides of the network merge after each skip connection that ends at the decoder blocks on each level, similar to the implementation with nnUNet as the backbone network.

Table 2 shows the performance of using the two backbone networks on the original dataset, and Table 3 shows the performance comparisons when excluding all FN lesions. The AtPCa-Net(nnUNet) and AtPCa-Net(nnUNet++) represents for AtPCa-Net using nnUNet and nnUNet++ as a backbone network, respectively. From both the Table 2 and the Table 3, we can observe that both nnUNet-based AtPCa-Net and nnUNet++-based AtPCa-Net achieved better detection and classification performance than when only using the nnUNet or nnUNet++, respectively (p<0.05). This indicates the generalizable potential of applying the proposed anatomical-aware design with different backbone networks. From Table 2, we see the nnUNet-based AtPCa-Net performs better on patient-level classification and also achieves higher sensitivities at 0.5/1 FP predictions per patient. When the rate of FP predictions per patient raises to 2/2.5 FP predictions per patient, the nnUNet++-based AtPCa-Net achieves higher sensitivities. In Table 3, nnUNet-based AtPCa-Net outperforms the nnUNet++-based AtPCa-Net on all situations, except similar on patient-level classification AUC and sensitivity at 1.5 FP predictions per patient. We select nnUNet as the backbone as it outperforms the nnUNet++-based AtPCa-Net in the majority of situations and represents the nnUNet-based AtPCa-Net as AtPCa-Net if without any other descriptions in this paper.

**Table 2:** Model performance comparisons using different backbone networks.

Models	Patient classification AUC (95% CI)	csPCa detection sensitivity (95% CI)				
		0.5 FP/Patient	1 FP/Patient	1.5 FP/Patient	2 FP/Patient	2.5 FP/Patient
nnUNet	0.843 (0.803, 0.883)	0.634 (0.571, 0.697)	0.707 (0.651, 0.763)	0.736 (0.684, 0.788)	0.760 (0.707, 0.813)	0.789 (0.735, 0.843)
nnUNet++	0.867 (0.832, 0.902)	0.626 (0.554, 0.698)	0.695 (0.632, 0.758)	0.756 (0.698, 0.814)	0.781 (0.724, 0.838)	0.817 (0.769, 0.865)
AtPCa-Net(nnUNet)	**0.880 (0.846, 0.914)**	**0.675 (0.620, 0.730)**	**0.728 (0.674, 0.782)**	**0.772 (0.716, 0.828)**	0.793 (0.741, 0.845)	0.809 (0.757, 0.861)
AtPCa-Net(nnUNet++)	0.877 (0.842, 0.912)	0.662 (0.601, 0.723)	0.719 (0.662, 0.776)	**0.772 (0.719, 0.825)**	**0.809 (0.757, 0.861)**	**0.821 (0.771, 0.871)**

**Table 3:** Model performance comparisons using different backbone networks after excluding FN lesions.

Models	Patient classification AUC (95%CI)	csPCa detection sensitivity (95%CI)				
		0.5 FP/Patient	1 FP/Patient	1.5 FP/Patient	2 FP/Patient	2.5 FP/Patient
nnUNet	0.873 (0.810, 0.936)	0.783 (0.720, 0.846)	0.862 (0.811, 0.913)	0.889 (0.845, 0.933)	0.915 (0.880, 0.950)	0.921 (0.885, 0.957)
nnUNet++	0.876 (0.839, 0.913)	0.762 (0.697, 0.827)	0.836 (0.783, 0.889)	0.884 (0.836, 0.932)	0.905 (0.863, 0.947)	0.921 (0.879, 0.963)
AtPCa-Net(nnUNet)	**0.898 (0.866, 0.930)**	**0.825 (0.767, 0.883)**	**0.873 (0.822, 0.924)**	**0.900 (0.856, 0.944)**	**0.931 (0.890, 0.972)**	**0.931 (0.891, 0.971)**
AtPCa-Net(nnUNet++)	**0.898 (0.866, 0.930)**	0.820 (0.765, 0.875)	0.852 (0.800, 0.904)	**0.900 (0.855, 0.945)**	0.921 (0.882, 0.960)	0.926 (0.890, 0.962)

#### Ablation study

We conduct ablation studies to discover the importance of each component of the proposed AtPCa-Net, shown in Table 4. We can observe that either modifying the Focal Loss to the ZL or modifying the network architecture to be in the symmetric-aware architecture improves the performance on both per-patient level classification and csPCa detection. When all the components are included, which formats the proposed AtPCa-Net, it outperforms all other situations when only partial components are included, showing the superiority of our proposed method and the usefulness of integrating all the mentioned prostate anatomical-related prior into the model.

**Table 4:** Ablation study of effects of including/excluding components of the AtPCa-Net.

Components		Patient classification AUC (95%CI)	csPCa detection sensitivity (95%CI)				
Sym-aware	Zonal loss		0.5 FP/Patient	1 FP/Patient	1.5 FP/Patient	2 FP/Patient	2.5 FP/Patient
		0.843 (0.803, 0.883)	0.634 (0.571, 0.697)	0.707 (0.651, 0.763)	0.736 (0.684, 0.788)	0.760 (0.707, 0.813)	0.789 (0.735, 0.843)
$\checkmark$		0.878 (0.843, 0.913)	0.650 (0.586, 0.714)	0.707 (0.643, 0.771)	0.756 (0.693, 0.819)	0.785 (0.729, 0.841)	0.789 (0.731, 0.847)
	$\checkmark$	0.854 (0.817, 0.891)	0.642 (0.580, 0.704)	0.707 (0.651, 0.663)	0.744 (0.690, 0.798)	0.781 (0.729, 0.833)	0.793 (0.740, 0.846)
$\checkmark$	$\checkmark$	**0.880 (0.846, 0.914)**	**0.675 (0.620, 0.730)**	**0.728 (0.674, 0.782)**	**0.772 (0.716, 0.828)**	**0.793 (0.741, 0.845)**	**0.809 (0.757, 0.861)**

**Table 5:** Patient-level classification and csPCa detection performance comparisons of different models without FN lesions.

Models	Patient classification AUC (95%CI)	csPCa detection sensitivity (95%CI)				
		0.5 FP/Patient	1 FP/Patient	1.5 FP/Patient	2 FP/Patient	2.5 FP/Patient
nnUNet	0.873 (0.810, 0.936)	0.783 (0.720, 0.846)	0.862 (0.811, 0.913)	0.889 (0.845, 0.933)	0.915 (0.880, 0.950)	0.921 (0.885, 0.957)
ResidualUNet	0.844 (0.803, 0.885)	0.689 (0.610, 0.768)	0.762 (0.686, 0.838)	0.834 (0.769, 0.899)	0.887 (0.835, 0.939)	0.901 (0.853, 0.949)
VNet	0.861 (0.823, 0.899)	0.698 (0.628, 0.768)	0.799 (0.736, 0.862)	0.841 (0.785, 0.897)	0.862 (0.812, 0.912)	0.884 (0.840, 0.928)
SEResUNet	0.860 (0.822, 0.898)	0.725 (0.658, 0.792)	0.799 (0.742, 0.856)	0.852 (0.803, 0.901)	0.884 (0.838, 0.930)	0.900 (0.857, 0.943)
VoxResNet	0.874 (0.841, 0.907)	0.683 (0.616, 0.750)	0.773 (0.713, 0.833)	0.815 (0.762, 0.868)	0.836 (0.784, 0.888)	0.884 (0.836, 0.932)
AttentionUNet	0.857 (0.819, 0.895)	0.735 (0.666, 0.804)	0.820 (0.767, 0.873)	0.857 (0.806, 0.908)	0.884 (0.832, 0.936)	0.910 (0.869, 0.951)
UNETR	0.873 (0.836, 0.910)	0.735 (0.668, 0.802)	0.831 (0.774, 0.888)	0.862 (0.813, 0.911)	0.889 (0.841, 0.937)	0.905 (0.862, 0.948)
AtPCa-Net(Proposed)	**0.898 (0.866, 0.930)**	**0.825 (0.767, 0.883)**	**0.873 (0.822, 0.924)**	**0.900 (0.856, 0.944)**	**0.931 (0.890, 0.972)**	**0.931 (0.891, 0.971)**

## Discussion

Our study demonstrated that the anatomical-aware designs, specifically the symmetric-aware architecture and the ZL, of the AtPCa-Net can help improve the csPCa detection performance and also patient-level classification results. We attribute the improvements in our model not only to the zonal-related knowledge learned under the guidance of the anatomically-aware ZL but also to the ability to reduce FP, which is a direct result of our symmetric-aware network architecture design.

The proposed anatomical-aware designs in AtPCa-Net help improve model performance on both csPCa detection and patient-level classification. The ZL shows its effectiveness by taking the lesion appearance differences on mpMRI images in different prostate zones into consideration. There are several approaches^5,6^ trying to utilize the zonal information by stacking the zonal mask as part of input together with the CE loss function and have shown improvement in model performance. However, PCa lesions located in different prostate regions are treated identically by using the CE loss, ignoring the essential anatomical information related to PCa’s zonal appearance differences. By using the ZL, an additional anatomical-aware constraint is added, and the zonal masks are further utilized. In addition, the symmetric-aware architecture of the AtPCa-Net helps reduce the FP predictions that are related closely to other normal prostate tissue with similar visual appearances as the PCa lesions on mpMRI, like BPH and CZ. The symmetric nature of the proposed network design helps distinguish the differences between the asymmetric patterns of PCa and the symmetric patterns of other normal prostate tissue. We can see that the integration of both the anatomical-aware designs, the ZL and the symmetric-aware architecture, helps improve the model performance more compared with the situations when including each individual design only, with 4.1%/2.1%/3.6%/3.3%/2.0% sensitivity per 0.5/1/1.5/2/2.5 FP/Patient and 3.7% AUC improvements.

In this study, all patients with csPCa are confirmed by the WMHP results. Different from some of the existing studies using results confirmed by prostate biopsies^4,5,37^, our WMHP dataset has the retrospective annotations for MR-visible FN lesions that are prospectively missed. The model performance regarding the FN lesions is important as the missing lesions or underestimation of the PCa’s volume and significance could result in inadequate therapy and consequently undesired oncologic outcomes^32,33^. Although both Tables 1 and 5 show the consistent superiority of the proposed AtPCa-Net compared with other models, it also reveals that all model performances dropped on both ROC and FROC measurements when including FN lesions compared with the situation when all FN lesions are excluded. The results highlight the challenges in automatically identifying FN csPCa lesions via deep-learning models, which aligns with the observations from existing study about the difficulty to identify FN lesions in clinical practice^32^. FN lesions are typically tiny and sometimes might be affected by the spatial resolution of the MRI imaging, making it hard to be detected^32^. Future studies could potentially be conducted regarding how to build an effective automatic csPCa detection model focusing on issues related to FN csPCa lesions, in conjunction with advancements MR technology to enhance the resolution of mpMRI.

We also evaluated the proposed AtPCa-Net on patients cohorts grouped by different prostate-specific antigen density (PSAD) level. The PSAD level is one of the clinical factors indicating the level of potential risk of patients having PCa^49,50^. The results can be found in Table S2 and Table S3 in Supplementary Information. In all, we showed the csPCa detection and patient-level classification performances of the proposed AtPCa-Net on patient cohorts grouped by cut-off PSAD level of 0.15 ng/ml/ml and 0.20 ng/ml/ml, which used as recommended thresholds for evaluating the risk of patients having PCa in existing studies^49,50^. The results exhibited the proposed AtPCa-Net performed better in patient cohort with higher PSAD compared with the cohort with lower PSAD in both cut-off settings. Further improvement could be made to improve the model performance when integrating the clinical information with the DL model design. For example, the DL model may be able to capture the risk for the patient having csPCa by the imported PSAD level, and then learn to enhance the prediction efficacy accordingly. Collecting potential related clinical and demographic information and discovering how to effectively integrating them with the DL model designs could be our future research directions.

Several limitations exist in the study. The model evaluations might be affected by the fact that the WMHP dataset was collected from a single institution and with MR machines from a single vendor. In the future, we will expand the WMHP dataset with multi-center collaborations and multi-vendor data, improving the diversity of the dataset with multiple clinical settings and patient demographics, and validate the proposed model’s generalizability and further solidify our findings. In addition, the real-world diagnosis of PCa generally integrates radiological findings together with clinical test results and demographic information^2,3^. However, just like other existing studies^4–10,23^, our study is limited on only utilizing information from mpMRI images. Potential performance improvements could be achieved if including clinical test results and demographic information in the csPCa detection model design, since they have shown the ability to improve model performance compared with using imaging information only in other computer-aided disease diagnosis studies^51–55^. In addition, due to the limit role played by the DCE imaging in the clinical practice, we excluded the DCE imaging from the model design, like other existing studies with the same research objectives^51–55^. As the DCE imaging can provide microvascular structure information, it could also potentially contribute to improved PCa diagnosis by providing imaging information from another perspectives^3^. The integration of clinical information and radiological findings, and the inclusion of the DCE imaging could be our future research directions.

We have shown that by taking PCa-specific anatomical priors into consideration, the PCa detection model improves its performance on both csPCa detection and patient-level classification. We believe the advantage comes from the key ideas of fusing the anatomical-related clinical priors into the loss function and network architecture design, which can better guide model training. The achievement could potentially influence the future designs of the DL-based PCa detection models on how the anatomical priors could help enhance the performance when integrating with DL model designs. We hypothesize that integrating the disease-specific anatomical-related knowledge into the model design could also potentially improve the model performance for other diseases, which could be a future research direction.

## Conclusions

We have demonstrated that by integrating anatomical priors into the deep learning network architecture design, the model efficacy is enhanced on both clinically significant prostate cancer (csPCa) detection and patient-level classification. Adopted from the clinical interpretation, the anatomical priors are carefully achieved by a symmetric-aware architecture design and the Zonal Loss (ZL), which format the proposed 3D anatomical-aware prostate cancer detection network (AtPCa-Net). Our experiments show that the model performance improves when either symmetric-related anatomical priors or zonal appearance differences of PCa are considered, with the best results achieved when the model incorporates both information. The proposed AtPCa-Net shows superior performance to other baseline models in both csPCa detection and patient-level classification, and shows the potential to further reduce the number of unnecessary biopsies may be caused by using current DL models. Our approach also reveals the potential flexibility of the anatomical-aware designs as they can improve the model performance with different backbone networks. How to generalize the anatomical-aware design idea to other specific diseases and how to integrate the design with clinical test results and demographic information could be our future research directions.

### Supplementary Information

Supplementary Tables.

## Data Availability

The dataset collected from our institution is currently not publicly available, since the IRB only approves its use for internal usage. The data might be available for research purposes on reasonable request or institutional collaborations. Please contact the corresponding author for any dataset-specific requests.
