# Supplementary material for: AtPCa-Net: anatomical-aware prostate cancer detection network on multi-parametric MRI
Source: Sci Rep. 2024 Mar 8;14:5740. doi: 10.1038/s41598-024-56405-7 (PMC10923873; doi:10.1038/s41598-024-56405-7)
Supplement: Supplementary file 1 — Supplementary Tables. [file 41598_2024_56405_MOESM1_ESM.pdf]

AtPCa-Net: Anatomical-aware Prostate Cancer Detection Network on Multi-parametric MRI

Haoxin Zheng [*,1,2], *Alex Ling-Yu Hung* [1,2], *Qi Miao* [1], *Weinan Song*[3], *Fabien Scalzo* [2,4], *Steven S. Raman* [1], *Kai Zhao* [1], *Kyunghyun Sung* [1]

[1] Radiological Sciences, University of California – Los Angeles, Los Angeles, CA 90095, USA

[2] Computer Science, University of California – Los Angeles, Los Angeles, CA 90095, USA

[3] Electrical and Computer Engineering, University of California – Los Angeles, Los Angeles, CA 90095, USA

[4] Seaver College, Pepperdine University, Malibu, CA 90263, USA

*Corresponding author: Haoxin Zheng, haoxinzheng@g.ucla.edu

Supplementary Information

Table S1: Experiments for different methods of importing symmetric information into the DL model. Patient-level classification and csPCa detection performance comparisons between models that 1) importing original and flipped mpMRI images separately to two share-weighted encoders (note as Siamese), and 2) importing stacked original and flipped mpMRI images to a single encoder as input (note as Flipped). The ZL stands for the proposed Zonal Loss.

Models	Patient Classification AUC (95%CI)	csPCa Detection Sensitivity (95% CI)				
		0.5 FP/Patient	1 FP/Patient	1.5 FP/Patient	2 FP/Patient	2.5 FP/Patient
Flipped	0.854 (0.813, 0.892)	0.634 (0.570, 0.696)	0.699 (0.637, 0.756)	0.740 (0.681, 0.799)	0.768 (0.712, 0.826)	0.785 (0.729, 0.840)
Siamese	0.878 (0.843, 0.913)	0.650 (0.586, 0.714)	0.707 (0.643, 0.771)	0.756 (0.693, 0.819)	0.785 (0.729, 0.841)	0.789 (0.731, 0.847)
Flipped + ZL	0.870 (0.831, 0.906)	0.655 (0.593, 0.710)	0.699 (0.643, 0.758)	0.744 (0.693, 0.797)	0.768 (0.717, 0.820)	0.785 (0.734, 0.833)
Siamese + ZL	0.880 (0.846, 0.914)	0.675 (0.620, 0.730)	0.728 (0.674, 0.782)	0.772 (0.716, 0.828)	0.793 (0.741, 0.845)	0.809 (0.757, 0.861)

The results showed not only the "Siamese" model performed better than the "Flipped" model in all measurements, but also, by adding the proposed ZL, the "Siamese+ZL" performed better than the "Flipped+ZL". This indicated that importing original and flipped mpMRI images separately to two share-weighted encoders could be a better way to import symmetric information compared with importing stacked original and flipped mpMRI images to a single encoder as input.

Table S2: Patient-level classification and csPCa detection performance comparisons using the proposed AtPCa-Net on patients with prostate-specific antigen density (PSAD) level above 0.15 ng/ml/ml and below 0.15 ng/ml/ml.

Setting	Patient Classification AUC (95%CI)	csPCa Detection Sensitivity (95% CI)				
		0.5 FP/Patient	1 FP/Patient	1.5 FP/Patient	2 FP/Patient	2.5 FP/Patient
PSAD >= 0.15 ng/ml/ml	0.923 (0.869, 0.966)	0.780 (0.683, 0.881)	0.840 (0.750, 0.936)	0.900 (0.820, 0.976)	0.900 (0.820, 0.976)	0.900 (0.820, 0.976)
PSAD < 0.15 ng/ml/ml	0.841 (0.738, 0.925)	0.674 (0.519, 0.825)	0.744 (0.587, 0.881)	0.814 (0.667, 0.950)	0.837 (0.719, 0.950)	0.884 (0.776, 0.977)

Table S3: Patient-level classification and csPCa detection performance comparisons using the proposed AtPCa-Net on patients with prostate-specific antigen density (PSAD) level above 0.20 ng/ml/ml and below 0.20 ng/ml/ml.

Setting	Patient Classification AUC (95%CI)	csPCa Detection Sensitivity (95% CI)				
		0.5 FP/Patient	1 FP/Patient	1.5 FP/Patient	2 FP/Patient	2.5 FP/Patient
PSAD >= 0.20 ng/ml/ml	0.924 (0.862, 0.970)	0.800 (0.667, 0.926)	0.833 (0.706, 0.957)	0.900 (0.780, 1.000)	0.900 (0.780, 1.000)	0.900 (0.780, 1.000)
PSAD < 0.20 ng/ml/ml	0.868 (0.785, 0.934)	0.698 (0.574, 0.820)	0.778 (0.660, 0.885)	0.825 (0.714, 0.927)	0.841 (0.732, 0.941)	0.873 (0.787, 0.952)

The results from Table S2 and Table S3 exhibited that the proposed AtPCa-Net showed better performance on both detection and classification in patient cohort with higher PSAD compared with the cohort with lower PSAD, in both situations with cut-off PSAD level of 0.15 ng/ml/ml and 0.20 ng/ml/ml. This indicated that the proposed model generated relatively more FP predictions for patients with low risk of having csPCa. Further improvement could be made to improve the model performance when integrating the clinical information with the DL model design. For example, the DL model may be able to capture the risk for the patient having csPCa by the imported PSAD level, and then learn to enhance the prediction efficacy accordingly. Collecting potential related clinical and demographic information and discovering how to effectively integrating them with the DL model designs could be our future research directions.
